# Specific Medical Conditions Are Associated with Unique Behavioral Profiles in Autism Spectrum Disorders

**DOI:** 10.3389/fnins.2016.00410

**Published:** 2016-09-22

**Authors:** Ditza A. Zachor, Esther Ben-Itzchak

**Affiliations:** ^1^Sackler Faculty of Medicine, Tel Aviv UniversityTel Aviv, Israel; ^2^Department of Pediatrics, The Autism Center, Assaf Harofeh Medical CenterZerifin, Israel; ^3^Department of Communication Disorders, Ariel UniversityAriel, Israel

**Keywords:** autism spectrum disorders (ASD), microcephaly, macrocephaly, developmental regression, food selectivity, sleep problems, cognition, autism severity

## Abstract

Autism spectrum disorder (ASD) is a heterogeneous group of disorders which occurs with numerous medical conditions. In previous research, subtyping in ASD has been based mostly on cognitive ability and ASD symptom severity. The aim of the current study was to investigate whether specific medical conditions in ASD are associated with unique behavioral profiles. The medical conditions included in the study were macrocephaly, microcephaly, developmental regression, food selectivity, and sleep problems. The behavioral profile was composed of cognitive ability, adaptive skills, and autism severity, and was examined in each of the aforementioned medical conditions. The study population included 1224 participants, 1043 males and 181 females (M:F ratio = 5.8:1) with a mean age of 49.9 m (SD = 29.4) diagnosed with ASD using standardized tests. Groups with and without the specific medical conditions were compared on the behavioral measures. Developmental regression was present in 19% of the population and showed a more severe clinical presentation, with lower cognitive abilities, more severe ASD symptoms, and more impaired adaptive functioning. Microcephaly was observed in 6.3% of the population and was characterized by a lower cognitive ability and more impaired adaptive functioning in comparison to the normative head circumference (HC) group. Severe food selectivity was found in 9.8% and severe sleep problems in 5.1% of the ASD population. The food selectivity and sleep problem subgroups, both showed more severe autism symptoms only as described by the parents, but not per the professional assessment, and more impaired adaptive skills. Macrocephaly was observed in 7.9% of the ASD population and did not differ from the normative HC group in any of the examined behavioral measures. Based on these findings, two unique medical-behavioral subtypes in ASD that affect inherited traits of cognition and/or autism severity were suggested. The microcephaly phenotype occurred with more impaired cognition and the developmental regression phenotype with widespread, more severe impairments in cognition and autism severity. In contrast, severe food selectivity and sleep problems represent only comorbidities to ASD that affect functioning. Defining specific subgroups in ASD with a unique biological signature and specific behavioral phenotypes may help future genetic and neuroscience research.

## Introduction

Autism spectrum disorder (ASD) is a heterogeneous group of disorders which, in addition to its core symptoms, occurs with numerous medical and behavioral comorbidities and conditions. Therefore, there is great variability in the clinical manifestations, which may suggest different neurobiological and genetic etiologies.

In previous research, subtyping in ASD has been based on a variety of factors. Several studies have used autism symptom severity as a basis for subtyping, including severity of social communication deficit and level of restricted and repetitive behaviors (RRB) (Ingram et al., 2008; Wiggins et al., 2012; Georgiades et al., 2013). Others have used verbal and non-verbal cognitive ability for subgrouping. Individuals with ASD with and without intellectual disabilities differed in symptom severity and later outcomes (Sheinkopf and Siegel, 1998; Lord et al., 2006; Ben-Itzchak et al., 2008, 2014; Grzadzinski et al., 2013). In addition, cognitive profiles based on the discrepancy between verbal and non-verbal skills have been identified (Joseph et al., 2002; Munson et al., 2008).

Several medical conditions have frequently been described as co-occurring with ASD, including: macro—and microcephaly, developmental regression, feeding and sleep problems (Coury et al., 2012; Ben-Itzchak et al., 2013a). Research so far has not addressed the question of whether these medical conditions occur in specific ASD subtypes. Medical phenotypes can potentially be used as biological variables to define specific endophenotypes in ASD.

Accelerated head growth in early childhood, resulting in a relatively enlarged head circumference, has been reported in numerous studies. An increased prevalence (14–34%) of macrocephaly, head circumference ≥97%, has been described in children with ASD (Sacco et al., 2007, 2010; Ben-Itzchak et al., 2013a; Grandgeorge et al., 2013). It is hypothesized that in children with ASD, the brain undergoes an abnormal growth trajectory that includes a period of early overgrowth. This theory is supported by neuroimaging and neuropathological findings (Courchesne et al., 2003, 2007; Mraz et al., 2007; Webb et al., 2007). Whether macrocephaly is associated with specific clinical presentation in ASD has been inconsistently reported in previous studies. Some have described a higher level of functioning in children with ASD and macrocephaly in comparison to those with normative head circumference (Aylward et al., 2002; Courchesne and Pierce, 2005; Sacco et al., 2007). Larger head circumference percentiles were found in children with ASD and special abilities, as compared to those without special abilities (Ben-Itzchak et al., 2013b). Other studies have found no correlation between head circumference and cognitive abilities (Gillberg and deSouza, 2002; Deutsch and Joseph, 2003; Ben-Itzchak and Zachor, 2007).

Although macrocephaly has been widely explored, microcephaly is less well researched in individuals with ASD. Previous studies have reported an increased prevalence of microcephaly between 5.9 and 15.1% (Fombonne et al., 1999; Miles et al., 2005; Ben-Itzchak et al., 2013a) in comparison to the 3% reported in the general population. This condition was more frequent among girls (Miles et al., 2005; Ben-Itzchak et al., 2013a) and among participants with intellectual disabilities (Fombonne et al., 1999).

A unique aspect of the developmental trajectory in ASD has been the occurrence of developmental regression, characterized by the loss of previously acquired skills. Studies have described an estimated prevalence of developmental regression of 15–30% in individuals with ASD (Baird et al., 2008; Parr et al., 2011; Ben-Itzchak et al., 2013a). Several studies reported that children with a history of developmental regression demonstrated more severe autism symptoms and more impaired cognition in comparison to population with ASD without a history of regression (Meilleur and Fombonne, 2009; Xi et al., 2010; Parr et al., 2011).

Food selectivity is one of the major eating problems described in ASD (Cermak et al., 2010). Previous studies on the prevalence of food selectivity in individuals with ASD have reported highly variable rates, ranging from 13 to 87% (Cornish, 1998; Klein and Nowak, 1999; Whiteley et al., 2000). Several studies have compared food selectivity in ASD populations to typically developing children (Schreck et al., 2004; Schmitt et al., 2008) and to children with developmental disabilities (Field et al., 2003; Williams et al., 2005; Dominick et al., 2007). Overall these studies found that children with ASD refused more foods and had less varied diets than did the other populations, both clinical and typically developing. This lack of variation in diet is often associated with an inadequate nutrition intake (Postorino et al., 2015). Food preference was affected by food texture (Dominick et al., 2007; Schmitt et al., 2008) and food presentation (Schreck and Williams, 2006). It has been suggested that food selectivity is affected by sensory processing problems, which are common in individuals with ASD (Williams et al., 2000; Kern et al., 2006). Food selectivity may also stem from tactile and oral hypersensitivities resulting from a more general problem in sensory modulation (reviewed in Marí-Bauset et al., 2014). The presence of food selectivity was associated with more severe autism symptoms based only on parental report, but not as observed by clinicians' assessments. ASD with food selectivity was associated with poorer non-verbal cognitive abilities, but no difference was found in adaptive skills in comparison to ASD subjects without food selectivity (Postorino et al., 2015).

Sleep problems are highly prevalent in ASD and are considered one of the most common co-morbid disorders (Couturier et al., 2005; Cotton and Richdale, 2006; Ming et al., 2008). Prevalence rates of sleep problems in ASD vary widely, ranging from 40 to 80% (Johnson et al., 2009) and are markedly higher than the 25–40% expected rate in the general population (Meltzer and Mindell, 2008; Reynolds and Malow, 2011). Chronic circadian rhythm sleep-wake cycle disturbances are common in ASD; however, when using a grading system to define the severity of the sleep problems, a lower rate of 13% was found for the ASD group, as compared with 5% for the typically developing populations (Krakowiak et al., 2008). Sleep serves many functions during early development, including brain growth, memory consolidation, and cognition (Stores and Wiggs, 1998). In the general population, sleep disturbances may impact the child's behavior, attention, cognition, and school performance (Gregory and Sadeh, 2012). Previous research has reported that sleep problems are associated with more severe core autism symptoms in social communication and repetitive behaviors, and in maladaptive behaviors like self-injury tantrums and aggression (reviewed in: Cohen et al., 2014; Herrmann, 2015). Parent-reported autism severity has been found to be the strongest predictor of sleep problems in children with ASD (Mayes and Calhoun, 2009). Certain core features of ASD have been associated with specific sleep problems, suggesting that sleep problems are inherently a part of the ASD diagnosis (Hollway et al., 2013). Children with ASD and sleep problems have lower adaptive functioning (Sikora et al., 2012), and lower performance scores on cognitive memory tasks as compared to young adults with ASD who do not experience sleep problems (Limoges et al., 2013). However, other studies that have looked at the association between sleep disorders and cognitive and adaptive skills have found that the subjects' cognitive level and adaptive functioning did not predict the severity of their sleep problems (Richdale and Prior, 1995; Krakowiak et al., 2008).

The aim of the current study was to investigate whether specific medical conditions in ASD are associated with unique behavioral profiles. Medical conditions included in the study were macrocephaly, microcephaly, developmental regression, food selectivity, and sleep problems. The behavioral profile was composed of cognitive ability, adaptive skills, and autism severity, and was examined in each of the aforementioned medical conditions.

## Materials and methods

### Participants

Participants who underwent a comprehensive assessment and were diagnosed with ASD at a tertiary autism center between the years 2001–2016 were included. Inclusion criteria were: having a diagnosis of ASD, falling in the age range of 15 month–12:0 year, and not having any known genetic syndromes. The final population included 1224 participants, 1043 males and 181 females (M:F ratio = 5.8:1) with a mean age of 49.9 m (SD = 29.4).

### Measures

The outcome measures for this study are described briefly below.

*Autism Diagnostic Interview-Revised (ADI–R):* A semi-structured interview administered to parents, designed to diagnose autism according to DSM-IV criteria (Rutter et al., 2003). For assessment of autism severity we used the ADI algorithm scores in social interaction, communication, and repetitive restricted behavior (RRB) domains. In the ADI-R scoring system, higher scores reflect more severe autism symptoms.

*Autism Diagnostic Observation Scales* (ADOS)–A semi-structured, interactive schedule designed to assess social and communicative functioning in individuals who may have an ASD. Only one of the modules was administered, depending on the examinee's age and/or expressive language (Lord et al., 1999). The ADOS total algorithm score was used for calculating the total severity score using the ADOS calibrated severity scales (CSS) (Gotham et al., 2009). The scores of each of the ADOS sub-domains, social affect (SA) and RRB, were used to calculate each sub-domain severity score using the new SA-CSS and RRB-CSS (Hus et al., 2014). In the ADOS scoring system, higher scores reflect more severe autism symptoms.

*Vineland Adaptive Behavior Scales* (VABS; Sparrow et al., 1984, 2005)–a standardized caregiver interview designed to assess adaptive behaviors in children from birth through 18 years of age. The VABS is organized into four sub-domains: Communication, Daily Living Skills, Socialization, and Motor Skills, each of which yield a standard score (mean of 100, SD of 15). In addition, the measure yields a total score, the Adaptive Behavior Composite (mean of 100, SD of 15). In the VABS, higher scores reflect better functioning.

*Head circumference (HC):* HC measurements were performed using standard methods (Deutsch and Farkas, 1994; Deutsch and Joseph, 2003) by a senior child neurologist and were plotted on normative head circumference growth charts and converted to percentile values (Nellhaus, 1968).

*Developmental regression* was based on the definition of loss within the ADI-R. The ADI-R requires that any loss be coded only if the skill was established initially for at least 3 months, and the loss of skill must have continued for at least 3 months. In this study, the occurrence of regression was explored using the coding of definite loss (score = 2) of specified skills in language, social engagement, constructive or imaginary play, or motor skills in the ADI-R.

*Food selectivity:* was defined according to parental reports during the medical evaluation and was graded as follows: 0 = eats normally, or has a few specific foods that he won't eat (especially fruits/vegetables); 1 = has more restrictions (will only eat certain food groups), fairly limited options; 2 = very limited options, and/or will not eat certain textures or certain colors. (For example, only eats white foods; only eats dry foods).

*Sleep problems:* were defined according to parental reports during the medical evaluation and were graded as follows: 0 = normal sleep behaviors, or occasional sleep disturbances; 1 = either wakes up regularly in the middle of the night (falls asleep again independently), or has difficulty falling asleep on a regular basis (at least 3 times per week); 2 = significant induction and sleep maintenance problems which interfere with family life and/or require medical treatment.

### Procedure

The study was conducted at a specialized autism center within a tertiary medical center that provides diagnosis and treatment services and is involved in research in the field of ASD. The evaluation included a neurological assessment and behavioral and cognitive evaluations. Assessments were conducted by a skilled interdisciplinary team. Pediatric neurologists obtained medical, developmental and familial histories from the parents and conducted a comprehensive neurological examination of all the participants.

The diagnosis of ASD was obtained by using two standardized tests, the Autism Diagnosis Interview-Revised (ADI-R) (Rutter et al., 2003) and the Autism Diagnosis Observation Schedule (ADOS) (Lord et al., 1999), and by meeting criteria for ASD based on DSM-IV (American Psychiatric Association, 2000) or DSM 5 (American Psychiatric Association, 2013) criteria, depending on the date of the evaluation. Data on ADI scores were available for 1194 participants and on ADOS severity scale scores for 1174 participants. All the professionals involved in the diagnostic process established reliability as required.

Cognitive and developmental abilities (IQ/DQ) were assessed using standardized cognitive assessments according to the child's age and language level. The following tests were used: The Mullen Scales of Early Learning (Mullen, 1995), Bayley Scales of Infant Development (Bayley, 1993); Wechsler Preschool and Primary Scale of Intelligence (Wechsler, 1989); Stanford-Binet Intelligence Scales (Thorndike et al., 1986); Kaufman Assessment Battery for Children-II (Kaufman and Kaufman, 1983); and Wechsler Intelligence Scale for Children IV (Wechsler, 2003). DQ/IQ scores were available for 758 participants.

Adaptive skills were assessed using the Vineland Adaptive Behavior Scales (VABS) (Sparrow et al., 1984) and were available for 937 participants.

The entire study population was classified according to the following medical phenotypes:

1. Head circumference (HC): HC data was available on 1194 participants. Three groups were defined based on the HC measurements: microcephaly HC ≤ 3 percentile, macrocephaly HC≥97 percentile, and a normative HC group between the 25 and 75th percentiles.
2. Developmental regression: the group included participants with a history of definite loss of specified skills (ADI-R items 11 and/or 20 score = 2).
3. Food selectivity: the group included participants with major food selectivity (score = 2). Data was available on 1188 participants.
4. Sleep problems: the group included participants with significant sleep problems (score = 2). Data was available on 1224 participants.

This research was approved by the IRB at the Medical Center as required.

### Data analysis

The dependent variables in this study included the following behavioral measures: autism severity measures (ADI-R social interaction, communication and RRB subdomains scores, ADOS-CSS in SA and RRB subdomain scores); cognitive ability (DQ/IQ scores); adaptive behavior (VABS communication, DLS, socialization and motor skills subdomain scores). The independent variables included the specific medical phenotypes: microcephaly, macrocephaly, developmental regression, eating problems and sleeping problems. To investigate the differences between the groups with and without the examined medical phenotype, a series of one way MANOVAs (for the ADOS-CSS, ADI-R and VABS subdomains scores) and ANOVAs (for DQ/IQ scores) were performed. The microcephaly group and the macrocephaly group were compared to the normative HC group (measures between 25 and 75th percentiles). The macrocephaly group differed in age from the normative HC group, and therefore age was controlled for when using ANOVA and MANCOVAs for this independent variable. Head circumference is expressed in percentiles based on normative data in large populations. Therefore, it was possible to compare the frequencies of microcephaly and macrocephaly percentile measures in the study population to the expected percentage in the general population (3%), Chi Square goodness-of-fit tests were used.

## Results

For each of the medical conditions, the frequency of occurrence in the ASD population was examined. Then, the groups with and without the specific medical conditions were compared for their clinical presentation in autism severity, cognitive ability and adaptive skills.

The Macrocephaly condition: of the ASD population that had data on HC, 89 (7.9%) had macrocephaly, 77 males and 12 females (6.4:1). This rate is significantly higher than the expected 3% in the general population [$\chi_{\left(1\right)}^{2}$ = 126.8, *p* < 0.001]. The macrocephaly group was significantly older than the normative HC group (Table 1) but did not differ significantly in their cognitive level (Table 1). The MANOVAs for the VABS scores [*F*_(4, 679)_ = 1.6, *p* = 0.182, *h*^2^ = 0.009] ADOS-CSS [*F*_(2, 831)_ = 0.4, *p* = 0.658, *h*^2^ = 0.001], and the ADI-R [*F*_(3, 857)_ = 0.8, *p* = 0.764, *h*^2^ = 0.001] while controlling for age, did not yield a significant group effect.

**Table 1:** **Mean and SD of cognitive scores, VABS scores and autism severity measures for the normative and macrocephaly groups**.

	**Normative HC**		**Macrocephaly**		***F***	***p***	**η^2^**
	***n***	***M(SD)***	***n***	***M(SD)***			
Age	781	49.1 (28.4)	89	65.2 (36.8)	24.0^*^	0.000	0.027
DQ/IQ	507	79.3 (21.7)	58	81.8 (24.9)	0.1	0.737	0.000
VABS	621		64				
Communication		80.3 (15.7)		78.9 (20.6)	1.1	0.282	0.002
DLS		76.6 (12.2)		73.8 (14.4)	3.1	0.078	0.005
Socialization		74.2 (10.9)		71.1 (13.2)	3.5	0.061	0.005
Motor skills		85.6 (13.8)		80.4 (15.1)	5.3^**^	0.022	0.008
ADOS-CSS	747		88				
SA		6.9 (2.2)		7.2 (2.2)	0.7	0.409	0.001
RRB		8.1 (1.7)		7.9 (2.2)	0.0	0.932	0.000
ADI-R	773		89				
Social interaction		14.2 (6.6)		14.2 (6.7)	0.2	0.659	0.000
Communication		10.4 (4.1)		11.1 (3.9)	1.1	0.303	0.001
RRB		4.9 (2.6)		5.3 (2.4)	0.0	0.871	0.000

** *p < 0.01*,

* *p < 0.05*.

Since the group with macrocephaly was significantly older than the group without macrocephaly, we examined the frequency of macrocephaly in 3 age ranges (15 month–2 year; 2:1–5 year; 5:1–12 year). A nonparametric analysis showed that the older the group, the higher the frequency of macrocephaly. The frequency of macrocephaly in the youngest age range was 6.7% (6 out of 218 participants), 7.3% in the middle age range (42 of 578 participants), and 12.2% in the oldest age range (41 of 337 participants) [$\chi_{\left(2\right)}^{2}$ = 16.8, *p* < 0.001].

The microcephaly condition: in the research population, 77 participants (6.3%) had microcephaly, among them 64 males and 13 females (4.9:1). This rate was significantly higher than the expected 3% in the general population [$\chi_{\left(1\right)}^{2}$ = 46.0, *p* < 0.001]. The MANOVA for the VABS scores [*F*_(4, 667)_ = 4.6, *p* = 0.001, *h*^2^ = 0.027] yielded a significant group effect. The autism severity scores in both the ADOS-CSS [*F*_(2, 811)_ = 0.3, *p* = 0.732, *h*^2^ = 0.001] and the ADI-R [*F*_(3, 836)_ = 0.7, *p* = 0.519, *h*^2^ = 0.003] did not yield a significant group effect. As shown in Table 2, the microcephaly group had lower DQ/IQ scores and lower VABS subdomains scores (communication, DLS, socialization, and motor skills) than the normative HC group.

**Table 2:** **Mean and SD of cognitive scores, VABS scores and autism severity measures for the normative and the microcephaly groups**.

	**Normative HC**		**Microcephaly**		***F***	***p***	**η^2^**
	***n***	***M(SD)***	***n***	***M(SD)***			
Age	772	49.1 (28.8)	78	44.5 (24.9)	1.8	0.176	0.002
DQ/IQ	507	79.6 (22.0)	47	67.7 (17.1)	13.0^***^	0.000	0.023
VABS	612		61				
Communication		80.3 (15.8)		73.8 (16.2)	9.5^**^	0.002	0.014
DLS		76.8 (12.0)		71.4 (13.6)	10.8^**^	0.001	0.016
Socialization		74.0 (10.7)		70.6 (10.8)	5.6^*^	0.019	0.008
Motor skills		85.8 (13.8)		78.5 (13.8)	15.3^***^	0.000	0.022
ADOS-CSS	739		76				
SA		6.9 (2.2)		6.9 (2.1)	0.1	0.759	0.000
RRB		8.1 (1.8)		8.0 (1.4)	0.3	0.559	0.000
ADI-R	765		76				
Social interaction		14.5 (10.0)		15.5 (7.2)	0.7	0.402	0.001
Communication		10.5 (4.1)		10.3 (3.5)	0.2	0.667	0.000
RRB		4.9 (2.5)		5.1 (2.8)	0.6	0.420	0.001

*** *p < 0.001*,

** *p < 0.01*,

* *p < 0.05*.

The developmental regression condition: in the research population, 230 participants (19.0%), including 186 males and 44 females (4.2:1), had a history of developmental regression. The MANOVAs for the VABS scores [*F*_(4, 924)_ = 9.4, *p* < 0.001, *h*^2^ = 0.014], the ADOS-CSS [*F*_(2, 1160)_ = 4.4, *p* = 0.012, *h*^2^ = 0.007] and the ADI-R [*F*_(3, 1189)_ = 17.5, *p* < 0.000, *h*^2^ = 0.042] yielded significant group effects. As shown in Table 3, the group with a history of developmental regression had lower DQ/IQ and VABS scores in all examined subdomains (communication, DLS, socialization, motor skills). In addition the group had higher autism severity scores in both of the autism severity measures. The group with developmental regression had higher ADOS-CSS-SA scores and higher ADI-R scores in all the examined subdomains (social interaction, communication and RRB), compared to the group without a history of developmental regression.

**Table 3:** **Mean and SD of cognitive scores, VABS scores and autism severity measures for the groups with and without a history of developmental regression**.

	**Regression**−		**Regression**+		***F***	***p***	**η^2^**
	***n***	***M(SD)***	***n***	***M(SD)***			
Age							
DQ/IQ	624	80.0 (22.1)	128	73.2 (19.9)	10.6^**^	0.001	0.014
VABS	757		173				
Communication		80.0 (15.8)		73.5 (16.7)	23.9^***^	0.000	0.025
DLS		76.2 (12.3)		72.6 (13.8)	11.6^**^	0.001	0.012
Socialization		74.1 (10.7)		69.6 (12.0)	24.4^***^	0.000	0.026
Motor skills		84.9 (13.9)		84.3 (15.5)	0.3	0.595	0.000
ADOS-CSS	943		219				
SA		6.9 (2.2)		7.3 (2.1)	8.1^**^	0.004	0.007
RRB		8.1 (1.7)		8.3 (1.8)	2.9	0.89	0.002
ADI-R	967		227				
Social interaction		13.9 (6.5)		18.4 (15.4)	47.1^***^	0.000	0.038
Communication		10.3 (4.0)		11.6 (3.9)	20.9^***^	0.000	0.017
RRB		4.8 (2.5)		5.4 (2.4)	9.0^**^	0.003	0.007

*** *p < 0.001*,

** *p < 0.01*.

The food selectivity condition: in the research population, 33.5% had food selectivity problems (score = 1 or 2). Of the ASD population, 116 participants (9.8%) had severe food selectivity (score = 2), including 98 males and 18 females (5.4:1). The MANOVAs for the VABS scores [*F*_(4, 912)_ = 4.7, *p* = 0.001, *h*^2^ = 0.020] and the ADI-R [*F*_(3, 1162)_ = 6.8, *p* < 0.000, *h*^2^ = 0.017] yielded significant group effects. The ADOS-CSS [*F*_(2, 1133)_ = 1.1, *p* = 0.324, *h*^2^ = 0.002] did not yield a significant group effect. As shown in Table 4, the group with food selectivity had lower VABS scores in all the examined subdomains (communication, DLS, socialization, motor skills) as compared to the group without food selectivity. In addition, the food selectivity group had higher autism severity scores in all the ADI-R subdomains (social interaction, communication, RRB) than the group with no food selectivity. The DQ/IQ scores did not differ between these two groups.

**Table 4:** **Mean and SD of cognitive scores, VABS scores and autism severity measures for the groups with and without food selectivity**.

	**Food selectivity−**		**Food selectivity+**		***F***	***p***	**η^2^**
	***n***	***M(SD)***	***n***	***M(SD)***			
Age	1072	49.4 (29.0)	116	53.3 (33.5)	1.8	0.181	0.002
DQ/IQ	691	78.9 (21.8)	58	76.9 (21.8)	0.5	0.491	0.001
VABS	842		75				
Communication		79.6 (16.0)		73.4 (15.7)	10.4^**^	0.001	0.011
DLS		76.2 (12.7)		70.0 (11.6)	16.4^***^	0.000	0.018
Socialization		73.8 (11.1)		68.7 (9.8)	15.1^***^	0.000	0.016
Motor skills		85.2 (14.1)		80.3 (15.0)	8.1^**^	0.005	0.009
ADOS-CSS	1024		113				
SA		6.9 (2.2)		7.2 (2.2)	2.2	0.138	0.002
RRB		8.1 (1.7)		8.2 (1.7)	0.5	0.488	0.000
ADI-R	1052		115				
Social interaction		14.3 (9.3)		16.7 (6.5)	7.3^**^	0.007	0.006
Communication		10.3 (4.0)		11.7 (3.5)	12.6^***^	0.000	0.011
RRB		4.8 (2.5)		5.8 (2.5)	13.9^***^	0.000	0.012

*** *p < 0.001*,

** *p < 0.01*.

The sleep problems condition: in the research population, 35% had sleep problems (scores = 1 or 2). Of the ASD population, 60 participants (5.1%) had severe sleep problems (score = 2), including 49 males and 11 females (4.4:1). The MANOVAs for the VABS scores [*F*_(4, 908)_ = 6.0, *p* < 0.001, *h*^2^ = 0.026] and the ADI-R [*F*_(3, 1153)_ = 9.2, *p* < 0.000, *h*^2^ = 0.023] yielded significant group effects. The ADOS-CSS [*F*_(2, 1121)_ = 0.8, *p* = 0.455, *h*^2^ = 0.001] did not yield a significant group effect. As shown in Table 5, the group with sleep problems had lower VABS scores in all the examined domains (communication, DLS, socialization, motor skills). In addition, the group with sleep problems had higher autism severity scores in all ADI-R subdomains (social interaction, communication, RRB) as compared to the group with no sleep problems. The DQ/IQ scores did not differ between the groups.

**Table 5:** **Mean and SD of cognitive scores, VABS scores and autism severity measures for the groups with and without sleep problems**.

	**Sleep problems−**		**Sleep problems+**		***F***	***p***	**η^2^**
	***n***	***M(SD)***	***n***	***M(SD)***			
Age	1116	49.9 (29.6)	60	49.6 (30.7)	0.0	0.941	0.000
DQ/IQ	709	78.9 (21.8)	36	76.6 (24.1)	0.4	0.545	0.000
VABS	862		52		6.0^***^	0.000	0.026
Communication		79.4 (16.0)		72.1 (15.2)	10.3^**^	0.001	0.011
DLS		76.1 (12.5)		69.4 (13.7)	13.6^***^	0.000	0.015
Socialization		73.8 (11.0)		66.6 (11.0)	21.2^***^	0.000	0.023
Motor skills		85.2 (13.9)		77.7 (16.6)	14.0^***^	0.000	0.015
ADOS-CSS	1067		58		0.8	0.455	0.001
SA		6.9 (2.2)		6.7 (2.2)	0.8	0.373	0.001
RRB		8.1 (1.7)		7.9 (1.7)	1.3	0.262	0.001
ADI-R	1099		59		9.2^***^	0.000	0.023
Social interaction		14.4 (9.1)		18.8 (6.8)	13.3^***^	0.000	0.011
Communication		10.4 (4.0)		11.6 (4.7)	5.2^*^	0.022	0.005
RRB		4.8 (2.5)		6.4 (2.8)	20.5^***^	0.000	0.017

*** *p < 0.001*,

** *p < 0.01*,

* *p < 0.05*.

## Discussion

The current study investigated whether specific medical conditions in ASD are associated with unique behavioral profiles. Developmental regression was present in 19% of the population. The developmental regression subgroup showed a more severe clinical presentation in all the examined domains, with lower cognitive abilities, more severe autism symptoms in both the professional assessment (ADOS) and the parental descriptions (ADI-R), and more impaired adaptive functioning. Microcephaly (head circumference ≤ 3%) was observed in 6.3% of the study population. The subgroup with microcephaly was characterized by a lower cognitive ability and more impaired adaptive functioning. Food selectivity was found in 9.8% and severe sleep problems in 5.1% of the ASD population. The food selectivity and sleep problem subgroups showed more severe autism symptoms only as described by the parents (ADI-R), but not by the professional assessment (ADOS). In addition, the subgroup with severe sleep problems showed more impaired adaptive skills than the group without severe sleep problems. Macrocephaly was observed in 7.9% of the ASD population, which was significantly more than expected. A higher percentage of macrocephaly was found with increasing age. The macrocephaly subgroup did not differ from the normative head circumference group in any of the examined behavioral measures (cognition, autism severity and adaptive behavior).

These findings suggest the existence of three unique clinical subgroups (microcephaly, developmental regression, and sleep problems/food selectivity). All three subgroups showed poorer adaptive skills in comparison to the research population without these specific medical characteristics. However, the source of these adaptive impairments seems to stem from a different origin in each subgroup. The microcephaly and developmental regression subgroups seem to represent different medical-behavioral phenotypes, with a potential neurobiological origin that affects inherited traits. In the microcephaly phenotype, cognitive abilities were impaired, and seemed to be the basis for poorer adaptive skills. The developmental regression phenotype may stems from extensive neurodevelopmental insult, which results in global impairments in cognition and more severe autism symptoms. These insults may be the basis for the poor adaptive skills in this subgroup.

The food selectivity and sleep problems subgroup did not show different cognitive abilities, nor did it differ in observed ASD symptom severity in comparison to populations without these problems. In this subgroup, it seems that the medical problems directly affected the severity of the adaptive skills, as well as the parental perception of autism severity. Severe food selectivity and sleep problems seem to be comorbid symptoms in ASD that adversely affect the child's functioning, and lead to a harsher parental perception of adaptive skills and autism severity.

Macrocephaly is associated with an enlarged head circumference and has been well documented in many studies in ASD. Here, we found an overall high frequency of macrocephaly in the examined population, which increased with the age of the participants. These findings support the relationship between abnormal head growth and the occurrence of ASD, but macrocephaly is probably not related to a unique behavioral phenotype.

Regarding developmental regression, the 19% prevalence found in this study is within the range previously reported (15–30%) (Baird et al., 2008; Parr et al., 2011; Ben-Itzchak et al., 2013a). In agreement with our results, several studies have noted that poor cognitive and language outcomes are more likely in individuals with ASD who regress (Parr et al., 2011). Several reports have noted that ASD symptomatology is more severe in individuals who have regressed (Meilleur and Fombonne, 2009; Parr et al., 2011). The current study also identified more severe autism symptoms on standardized observation results (ADOS) and in parental reports (ADI-R). In contrast, several studies found no significant differences in ASD symptom severity between groups with and without a history of developmental regression (Malhi and Singhi, 2012; Kern et al., 2014).

A greater prevalence of microcephaly, a head circumference below the third percentile, has been documented in ASD (Miles et al., 2000, 2005; Ben-Itzchak et al., 2013a). The current study confirmed these findings by reporting twice the expected frequency of microcephaly, as well as a correlation between microcephaly and more pronounced cognitive impairment. Only one previous study has described similar results. Miles et al. (2005) reported that a subgroup in ASD defined as ‘complex autism’ had microcephaly and/or significant dysmorphology, and showed a more impaired cognitive level than the ‘essential autism’ group. In general, the association between microcephaly and intellectual impairments has been well documented in the literature (Von der Hagen et al., 2014).

A second medical condition related to head circumference is macrocephaly. In agreement with previous studies, the current study found a higher overall prevalence of macrocephaly in ASD (7.9%), which is, however, lower than was previously reported (14–34%) (Sacco et al., 2007, 2010; Grandgeorge et al., 2013.) The current study's finding that the prevalence of macrocephaly increases with age provides a more nuanced perspective of this condition. This finding may be related to accelerated head growth in ASD during childhood, as previously described in the literature (Courchesne et al., 2003, 2007). In this study, macrocephaly was not associated with a specific behavioral phenotype, a finding which differs from some previous studies that described a higher level of functioning in children with ASD and macrocephaly in comparison to those with a normative head circumference (Aylward et al., 2002; Courchesne and Pierce, 2005; Sacco et al., 2007).

Food selectivity has been commonly described in ASD (13–87%). In this study, the prevalence of food selectivity was found in a third of the research population. Only one previous study has explored an association between food selectivity and distinctive clinical features in ASD (Postorino et al., 2015). In accordance with this study, we found that the presence of severe food selectivity was associated with more severe reported autism symptoms (ADI-R). However, the clinical assessments (ADOS) did not support differences in autism severity in populations with and without food selectivity. In the current research, adaptive skills were significantly impaired among children with severe food selectivity, unlike the findings in Postorino et al. (2015), which did not report on differences in adaptive skills.

Prevalence rates of sleep problems in ASD vary widely, ranging from 13 to 80% depending on the definition of the severity of sleep problems (Krakowiak et al., 2008; Johnson et al., 2009). In the current study, more than a third of the research population had sleep problems. Our findings confirm the results of several previous studies, which noted that sleep problems in ASD are associated with more severe autism symptoms as reported by parents (Mayes and Calhoun, 2009; Goldman et al., 2012; Park et al., 2012; Tudor et al., 2012). One study reported that ASD severity, as assessed by the ADOS, predicted sleep disturbances (Hollway and Aman, 2011). This was not confirmed in our study. In addition, several previous studies found that lower adaptive skills were more pronounced among children with ASD and sleep problems, which corresponds with the findings of the current study (Sikora et al., 2012; Taylor et al., 2012). Only a few studies have reported a positive association between intellectual disability and sleep problems (Hollway and Aman, 2011; Taylor et al., 2012). However, other studies have looked at the association between sleep disorders and cognitive and adaptive skills, and have found that the subjects' cognitive levels did not predict the severity of their sleep problems (Richdale and Prior, 1995; Krakowiak et al., 2008), which is similar to our findings. Several studies have reported findings similar to ours that subjects with ASD and sleep problems were associated with more impaired adaptive functioning (Hollway and Aman, 2011; Sikora et al., 2012; Taylor et al., 2012). Others did not find such an association (Richdale and Prior, 1995; Krakowiak et al., 2008).

Our findings suggest that the existence of severe food selectivity or severe sleep problems has a negative impact on the family life, and therefore parents perceive their children as having more severe autism symptoms and poorer functioning. It is worth noting that the direct and objective examination of cognitive ability and ASD severity by professional assessments is not affected by the presence of food selectivity or sleep problems. The difficulties encountered with feeding the child and a lack of sleep at night imposes a great burden on the family. The findings of this study strengthen the notion that the parental perception of their child's functioning is influenced by negative day-to-day experiences.

To summarize, the findings of this study point to the existence of two subtypes with specific biological markers that occur with inherited impairments. One subtype includes the microcephaly phenotype, which is accompanied by more impaired cognitive ability. The second subtype includes the developmental regression phenotype, which is accompanied by widespread, more severe impairments in cognition and autism severity. The medical food selectivity and sleep problems seem to be comorbid to ASD, and are associated with a more severe parental perception of the child's adaptive functioning and severity of autism symptoms. Macrocephaly was not associated with a specific phenotype and seems to be a feature of a subgroup with idiopathic ASD.

This study is innovative in its use of a comprehensive examination of different developmental domains in relation to specific medical problems to identify specific medical-behavioral subtypes. The study has several strengths. The ASD study population was large and well-characterized, with comprehensive medical backgrounds for the participants. Direct assessments of the participant and parental interview enabled us to receive different perspectives of the participants' characteristics. In addition, the definition of the medical problems was based on very stringent criteria.

One of the study's limitations was that not all the participants had all the examined variables. In addition, the background information provided and the severity of the food selectivity and sleep problems were based on parental reports and not on standardized measures.

The two medical-behavioral subtypes described in this study, the microcephaly and the developmental regression phenotypes, should be further investigated using advanced genetic, imaging, and neurobiological research. Future studies should explore other medical variables in relation to developmental and behavioral features of ASD. Defining specific subgroups with a unique biological signature that are associated with specific medical-behavioral phenotypes may help identify more homogenous groups within the heterogeneous ASD population.

## Author contributions

The authors DZ and EB tasks include substantial contributions to the conception or design of the work; the acquisition, analysis, and interpretation of data for the work; drafting the work and revising it critically for important intellectual content; final approval of the version to be published, and agreement to be accountable for all aspects of the work in ensuring that questions related to the accuracy or integrity of any part of the work are appropriately investigated and resolved.

### Conflict of interest statement

The authors declare that the research was conducted in the absence of any commercial or financial relationships that could be construed as a potential conflict of interest.
